# Brazilian Protocol for Sexually Transmitted Infections 2020: human papillomavirus (HPV) infection

**DOI:** 10.1590/0037-8682-790-2020

**Published:** 2021-05-17

**Authors:** Newton Sergio de Carvalho, Roberto José de Carvalho da Silva, Isabel Cristina do Val, Maria Luiza Bazzo, Mariângela Freitas da Silveira

**Affiliations:** 1 Universidade Federal do Paraná, Departamento de Tocoginecologia, Curitiba, PR, Brasil.; 2 Secretaria de Estado da Saúde, Centro de Referência e Treinamento DST/Aids, São Paulo, SP, Brasil.; 3 Universidade Federal Fluminense, Departamento Materno-Infantil, Niterói, RJ, Brasil.; 4 Universidade Federal de Santa Catarina, Programa de Pós-Graduação em Farmácia, Florianópolis, SC, Brasil.; 5 Universidade Federal de Pelotas, Programa de Pós-Graduação em Epidemiologia, Pelotas, RS, Brasil.

**Keywords:** Papillomavirus infections, Papillomaviridae, Condylomata acuminate, Warts

## Abstract

This article addresses human papillomavirus (HPV) infection, one of the topics covered by the Clinical Protocol and Therapeutic Guidelines for Comprehensive Care for People with Sexually Transmitted Infections, published by the Brazilian Ministry of Health. The Protocol and Guidelines have been developed based on scientific evidence and validated in discussions with specialists. This article presents epidemiological and clinical aspects and guidelines for health service managers and health workers about diagnosing and treating people with papillomavirus infection. This theme is a significant public health issue since it is the most prevalent sexually transmitted infection globally, capable of triggering the oncogenic process of cervical cancer and the possibility of anogenital warts. Important information is presented for gaining knowledge about HPV and action strategies for infection prevention and control, provision of quality care, and effective treatment of the disease.

## Highlighted excerpt

Infection by the human papillomavirus is the sexually transmitted infection of the highest contagion, superior to genital herpes and HIV.

## FOREWORD

The article addresses the infection by the human papillomavirus (HPV). This subject composes the Clinical Protocol and Therapeutic Guidelines (PCDT) for Comprehensive Care for People with Sexually Transmitted Infections (STI), published by the Health Surveillance Secretariat of the Ministry of Health. For the PCDT elaboration, a selection and analysis of the evidence available in the literature were performed, and examined by a panel of specialists. The National Committee approved the document for the Incorporation of Technologies in the Brazilian National Health System (Conitec)^1^ and updated it by the STI specialists team in 2020. 

## EPIDEMIOLOGICAL ASPECTS

HPV is a double-stranded, non-encapsulated DNA virus, a member of the Papillomaviridae family. It infects the squamous epithelium and can induce the formation of a wide variety of cutaneomucous lesions, especially in the anogenital region. More than 200 types of HPV are identified, of which approximately 40 affect the anogenital tract^2^.

The main form of HPV transmission is sexual activity of any kind, including the deposition of the virus in the fingers through genital contact and autoinoculation. Exceptionally, during labor, the formation of cutaneomucous lesions in newborns or recurrent laryngeal papillomatosis may occur. Transmission by fomites is rare. It is the most transmissible STI, greater than genital herpes and human immunodeficiency virus (HIV) infections^3^. The overall estimated risk for exposure to HPV infection is 15% to 25% for each new sexual partner^4^. Most sexually active people are likely to become infected at some point in their lives. At the beginning of the sexual activity and with a single partnership, women had a 28.5% risk of contracting HPV at the end of the first year and 50% at the end of the third year^4^. 

Generally, the infections are asymptomatic. Approximately 1% to 2% of the infected population will develop anogenital warts, and about 2% to 5% of women will undergo alterations in oncotic colpocytology. The prevalence of infection is higher in women under 30 years of age. In contrast, the vast majority of HPV infections in women (especially adolescents) have spontaneous resolution within an approximate period of up to 24 months^5^ ^-^ ^7^. The risk of acquiring a new HPV infection for women decreases with age; for men that risk does not change and remains high throughout life. However, once an HPV infection has been acquired, its average duration seems to be similar among men and women^8^. A study conducted in 26 Brazilian state capitals and the Federal District, which included 6,387 women with an average age of 21.6 years, identified a 53.6% prevalence of HPV^9^. 

## CLINICAL ASPECTS

The types of HPV that infect the anogenital tract can be of low or high oncogenic risk. The types belonging to the low-risk group (6, 11, 40, 42, 43, 44, 54, 61, 70, 72, and 81) often occur in benign lesions and low-grade squamous intraepithelial lesions. HPV types from the high-risk group (16, 18, 31, 33, 35, 39, 45, 51, 52, 56, 58, 59, 68, 73, and 82) or oncogenic are often associated with high-grade squamous intraepithelial lesions and carcinomas. It is important to stress that about 85% of low-grade lesions contain HPV of the oncogenic group^10^. Infection by a certain viral type does not prevent infection by other types of HPV, and multiple infections can occur. The average time between high-risk HPV infection and cervical cancer development is approximately 10 to 20 years. This period varies according to the type, the load, the virus persistence capacity, and the host’s immune status. Some individual characteristics are predisposing factors to the appearance of lesions, such as smoking, immune deficiencies (including those resulting from HIV infection), malnutrition, cancer, and the use of immunosuppressive drugs^11^.

**FIGURE 1:**
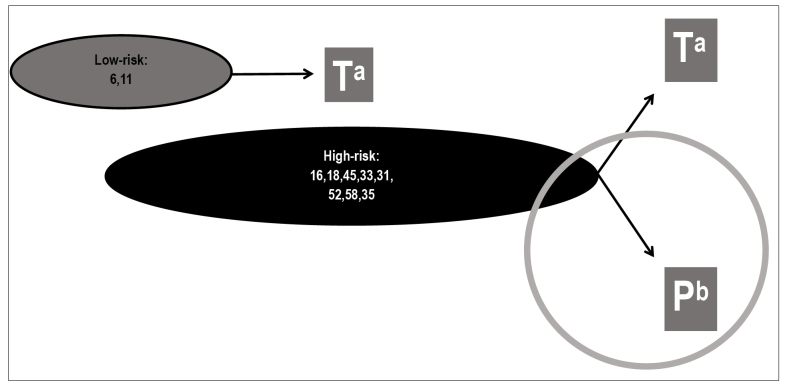
Prevalence of the human papillomavirus viral groups of low and high oncogenic risk and persistence capacity in the human organism.

In most people, HPV infection does not produce any clinical or subclinical manifestation. The latency period can vary from months to years. The prevalence of low and high-risk HPV viral groups in the population is demonstrated in Figure 1. Although the high-risk group is more prevalent (around 80%), only 20% of these infections persist and potentially cause high-grade lesions with cervical cancer progression. Percentages may vary according to age, geographic region surveyed, population profile, and methodology. In addition, there is a group of people who present simultaneously both low and high-risk HPV infection (about 30% of those infected), not included in Figure 1 ^12^.

a) T: transitory; b) P: persistent.

Subclinical manifestations of HPV can be detected by oncotic cytology, using magnifiers, coloring substances, and colposcopy, accompanied or not by biopsy. Lesions associated with low oncogenic risk viral types are generally low-grade squamous lesions, equivalent to the histopathological diagnosis of mild dysplasia or cervical intraepithelial neoplasia (CIN) grade 1. In contrast, lesions associated with high-risk oncogenic HPV infections are usually associated with high-grade squamous intraepithelial lesions and histopathological diagnosis of intraepithelial neoplasia grade 2 (CIN 2) or 3 (CIN 3). Besides the uterine cervix, other epithelia may suffer the oncogenic action of the virus, originating vaginal, vulvar, perianal, penile, and anal intraepithelial neoplasms^13^.

The clinically detectable manifestations induced by HPV are polymorphic and can be pointed (condyloma acuminatum), spiculated, with circumvolutions, or even flat. Their size varies from one millimeter to several centimeters. They can be single or multiple, flattened or papular, although in most cases, they are papillomatous. The lesions’ surface is opaque, velvety, or similar to that of cauliflower, and they may be of skin color, erythematous, or hyperpigmented. Generally, the lesions are asymptomatic and may be pruritic, painful, friable, or hemorrhaging^14^. 

In men, lesions occur most frequently on the inner fold of the foreskin, balanopreputial sulcus, or glans and may also be located on the skin of the penis and scrotum^15^. In women, they are usually seen on the vulva, vagina, and cervix. In both sexes, they may be found in the inguinal or perianal regions. Less frequently, lesions may be found in extragenital areas such as the conjunctiva and nasal, oral, and laryngeal mucosae^16^ ^,^ ^17^.

People living with HIV report a higher frequency of multiple infections, anogenital warts, intraepithelial lesions, and anogenital neoplasms due to HPV infection^18^ ^,^ ^19^. These data were confirmed in Brazil in a study by Miranda et al., and a 28.4% prevalence of high-risk HPV was found, in addition to the association with cytological abnormalities, age under 35, and illicit drug use^20^. 

## DIAGNOSIS

The diagnosis of anogenital warts is clinical. The biopsy for histopathological study must be performed when there is any doubt in the diagnosis (suspected neoplasms or other conditions), in the presence of atypical lesions or lesions that do not respond appropriately to treatment, and in suspicious or very voluminous lesions in people with immunodeficiencies. In the case of women with anogenital warts, a gynecological examination including cervical cytology is required for cervical cancer screening and, in the presence of cytological abnormalities, colposcopy and biopsy if necessary^21^. 

In the presence of anal lesions, a proctological examination with anoscopy and rectal touch, and even high-resolution anoscopy, would be ideal^22^. The cytological study of material collected from the anal canal is not yet indicated systematically; however, it may be indicated in special populations (men who have sex with men, people with receptive anal intercourse, and people with cancer or high-grade lesions) due to the increased incidence of anorectal cancer^23^. Tests for HPV typing are not recommended in clinical routine or in screening for asymptomatic people, especially adolescents, due to the high prevalence of HPV infection in this age group. These tests are important in screening for genital cancer, depending on the health system organization and its specific protocol.

Serological tests demonstrating natural antibodies, or after vaccination against HPV, lack practical applicability and have been used only in studies^24^. Therefore, for the diagnosis of HPV infection, besides clinically evaluating the condylomata (anogenital warts), it is essential to diagnose the pre-neoplastic lesions from the cervical cytology, molecular biology tests that demonstrates the presence of HPV and, if required, a colposcopy evaluation that defines the location of the lesions and guides the biopsy if necessary. The HPV test as a primary screening of pre-neoplastic lesions and its implementation in the Brazilian National Health System (SUS) is still under discussion^24^. 

## TREATMENT

The objective of the treatment of anogenital warts is to eradicate visible lesions (condylomata). However, there is no evidence that the treatments available so far modify the natural history of HPV infection. Even without treatment, the lesions can disappear, remain unchanged or increase in number and volume. Treatment of warts does not eliminate the HPV infection. The HPV DNA that remains in the infected cells can remain inactive (latent) for prolonged periods. The first episode or recurrence of symptoms may occur months or even years after the initial infection. Therefore, those who do not eliminate HPV can also transmit the virus, even after treatment or removal of the lesions. After initial elimination, the recurrence of warts is common: one year after treatment, approximately half of the cases develop new warts^25^. 

Several clinical and surgical treatments for anogenital warts are available, among which the following topical medicines: 60-80% trichloroacetic acid; 5% imiquimod cream; 10%-25% podophyllin solution; 0.5% podophyllotoxin solution; and 0.15% podophyllotoxin cream. As for the 0.5% podophyllotoxin solution, although not available in Brazil, neither at SUS nor at private health services, it must be highlighted for its benefits. The treatment with imiquimod or podophyllotoxin presents the convenience of self-application, although it requires guidance and rigorous monitoring due to the possibility of side effects. In turn, trichloroacetic acid and podophyllin must always be applied by the assistant physician and, likewise, with strict monitoring, due to the potential caustic effects. There is consensus that ablative techniques, such as electrotherapy, cryotherapy, and laser, are highly effective in treating anogenital warts. However, the evidence basis on the clinical efficacy and cost-effectiveness of the treatments used on anogenital warts is limited. The treatment must be individualized, considering the characteristics of the lesions, the availability of resources, the adverse effects, and the health professional’s experience. As this is a generally self-limiting disease, treatments that generate disfiguring scars should be avoided. Warts with soft, moist, and non-keratinized characteristics usually respond well to treatment with topical application of, for example, 5% imiquimod, 0.15% podophyllotoxin cream, or 60-80% trichloroacetic acid, depending on the type of lesion and skin characteristics. Physical ablative methods are more effective in the treatment of keratinized lesions^26^. Immunodeficiency situations, such as people who have undergone transplantation or people living with HIV, do not modify therapeutic recommendations. However, it is necessary to remember that individuals with these conditions tend to present a worse response to treatment, with greater proportions of relapse, demanding greater attention to the possibility of complications^27^.

The therapeutic option change should be considered when there is no significant improvement after three sessions or if warts do not disappear after six to eight treatment sessions. It is also possible to combine treatments, especially in complicated cases associated with immunosuppression, always with strict control of the normal tissues’ inflammatory effects^28^. 

There is also the possibility that anogenital warts will disappear without any treatment, especially in immunocompetent individuals^14^. Some people may opt for a waiting period before starting treatment. However, there is still uncertainty about the frequency of spontaneous resolution of lesions, with reports of untreated clearance rates up to 50% among affected people. In addition to increasing the risk of subsequent transmission, a delay in treatment may worsen warts, increases in their size, number, or affected area, particularly in people with impaired cellular immunity (pregnancy, HIV infection, or other immunosuppressions). A longer period may be needed to eradicate large or persistent warts^29^ ^,^ ^30^. Consideration should also be given to the psychosocial impact of manifestations of HPV infection. The health professional must guide clearly and objectively the person about the transmission and treatment of the disease^31^ ^,^ ^32^. 

It is also relevant to consider that the presence of genital warts acts as a “marker” for STI, as it is estimated that 20% of people with anogenital warts have a simultaneous STI, including chlamydia, HIV infection, or syphilis. Therefore, screening for other STIs should be performed. Chlamydia infection, when associated with HPV, not only complicates the treatment of the condylomata but also facilitates the progression of CIN lesions^33^. Immunosuppressive factors such as smoking habit, inadequate control of diabetes mellitus, and other diseases must be investigated and eliminated whenever possible, in addition to screening for precursor lesions to cancer and updating the Papanicolaou test. Condom use, in turn, shortens the process of viral elimination, promotes superior regression of CIN, and decreases the chance of condyloma relapse^34^. Screening and notification of sexual partners is not recommended, but the examination of current sexual partners should be considered, especially with the objective of screening for other STI^35^. 

**FIGURE 2:**
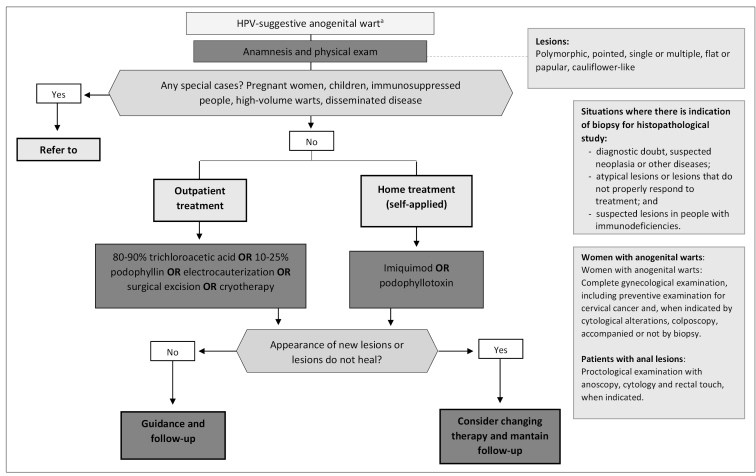
Recommendations for the treatment of anogenital warts, according to their morphology and distribution.

Figure 2 summarizes the recommendations for the treatment of anogenital warts according to their morphology and distribution.

a) HPV: human papillomavirus.

**Source:** adapted from the Clinical Protocol and Therapeutic Guidelines for Comprehensive Care for People with Sexually Transmitted Infections, 2020^1^.

## SURVEILLANCE, PREVENTION, AND CONTROL

Prophylactic vaccination is safe and effective in preventing HPV infection and its complications. There is evidence of the benefit of vaccination, both for individual and collective protection, with a reduction in benign and malignant lesions^36^ ^-^ ^38^. 

**FIGURE 3:**
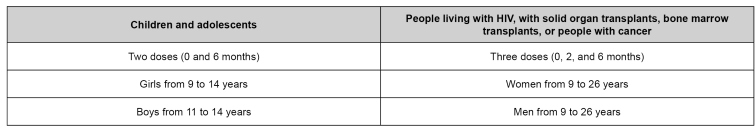
Indications for vaccination against human papillomavirus infection.

**Source:** Clinical Protocol and Therapeutic Guidelines for Comprehensive Care for People with Sexually Transmitted Infections, 2020^1^.

The vaccine is most effective in adolescents vaccinated before the first sexual contact, with an antibody production ten times higher than that found in the naturally acquired infection within two years. HPV vaccination does not lead to changes in sexual behavior among adolescents. Health professionals should indicate vaccination and promote the increase of vaccination coverage in the country^39^ ^-^ ^43^. Starting in 2014, the Ministry of Health expanded the National Immunization Schedule, introducing the quadrivalent HPV vaccine types 6 and 11 (low oncogenic risk) and 16 and 18 (high oncogenic risk). Figure 3 presents the indications for HPV vaccination^3^.

The HPV prophylactic vaccine must be a priority for SUS, considering its benefits related to immunogenicity, efficacy, and safety for the population’s health.
